# MicroRNA‐200c Promotes Epithelial–Mesenchymal Transition of Lens Epithelial Cells by Activating the Yes‐Associated Protein Signaling Pathway

**DOI:** 10.1155/joph/5384666

**Published:** 2026-07-14

**Authors:** Yin-Juan Wei, Yi-An-Zhu Liu, Ying Dai, Li Nan, Hui Song, Jun Li

**Affiliations:** ^1^ Department of Cataract, Tianjin Eye Hospital, Tianjin Key Lab of Ophthalmology and Visual Science, Tianjin, China, oio.cn; ^2^ Department of Ophthalmology, Affiliated Hospital of Jinggangshan University, Ji’an, China, jgsu.edu.cn; ^3^ Tianjin Eye Hospital, Tianjin Key Lab of Ophthalmology and Visual Science, Tianjin, China, oio.cn; ^4^ Department of Cataract, Tianjin Medical University Eye Hospital, School of Optometry & Eye Institute Tianjin Medical University, Tianjin, China, tmuec.com; ^5^ Department of Ophthalmology, Anxin County Hospital, Yong’an South Street, Anxin County, Xiongan New Area, China

## Abstract

**Purpose:**

The aim of the study is to investigate the role and possible mechanism of MicroRNA 200c (MiR‐200c) in epithelial–mesenchymal transition (EMT) of Posterior Capsule Opacification (PCO).

**Methods:**

Human lens epithelial cells (LECs) were treated with TGFβ2 to induce EMT as a model for PCO. The mRNA levels of miR‐200c and EMT markers were examined by real‐time quantitative polymerase chain reaction (qPCR). Protein expression levels and mRNA levels of EMT‐associated protein/transcriptional coactivators and Yes‐associated protein (YAP) were also compared between miR‐200c transfected and control cells by western blot analysis and qPCR. The scratch test was applied to detect the migration abilities of cells.

**Results:**

The expression level of miR‐200c was upregulated in TGFβ2‐induced EMT of LECs. Enhanced expression of miR‐200c via agomir transfection accelerated both cell migration and the transition to a mesenchymal phenotype. Conversely, miR‐200c inhibition using an antagomir effectively suppressed TGFβ2‐mediated EMT. Bioinformatics analysis revealed that YAP signaling could be positively regulated by miR‐200c, as evidenced by increased levels of YAP observed both in TGFβ2‐treated groups and transfected with miR‐200c agomir. Inhibition of Yap through the use of YAP‐TEAD Inhibitor 1 (Peptide 17) effectively prevented the upregulation of key EMT markers such as FN, vimentin, and transcription factor SNAIL during TGFβ2‐induced EMT.

**Conclusions:**

MiR‐200c promotes EMT and migration in LECs by activating the YAP signaling pathway. MiR‐200c may be used as a biomarker for the diagnosis or treatment in PCO.

**Key Messages**
• What is known:
◦ Epithelial–mesenchymal transition (EMT) plays a key role in the process of posterior capsule opacification. miR‐200 family has been proved to be associated with EMT in several malignant tumors and cancer.
• What is new:
◦ MiR‐200c plays a crucial role in the process of EMT and migration in lens epithelial cells (LECs), which is similar with malignant tumor cells.
◦ MiR‐200c is involved in the process of EMT in LECs through activating the YAP signaling pathway.

## 1. Background

Posterior Capsule Opacification (PCO), also known as after cataract, is one of the main complications after cataract surgery, which can significantly affect the long‐term visual quality of patients [1]. In pediatric cataract patients, the incidence of PCO reaches almost 100%, which could disrupt their visual development and lead to amblyopia [2]. There have been numerous studies regarding the pathogenesis of PCO; the mainstream theory is that surgical trauma causes blood–retinal barrier damage, increases the release of related inflammatory factors, and the residual lens epithelial cells (LECs) in the anterior capsule proliferate and migrate to the posterior capsule, undergo epithelial–mesenchymal transition (EMT), and eventually leads to PCO [3, 4].

TGFβ2 is considered one of the strongest cytokines that promotes EMT. Increasing evidence has demonstrated that TGFβ2 binds to TGFβ2 receptor types I and II, which then phosphorylates the intracellular signal transducers, Smad2 and Smad3. The heterotrimeric complex binds with Smad4 and migrates to the nucleus, leading to the activation of various genes associated with the EMT process [5, 6]. Intertwined with this process is the Hippo signaling pathway and its core effector, Yes‐associated protein (YAP). YAP acts as a sensor of the cellular microenvironment and mechanical stress; its nuclear translocation is critical for sustaining cell proliferation and fibrosis [7]. Importantly, the crosstalk between YAP and TGFβ2 signaling is essential for inducing the EMT. During the hepatic regenerative process, the hepatocyte nuclear accumulation of pSmad2 and YAP1 increased along with TGFβ2 expression [8]. Deleting Yap1 from hepatocytes caused diminished the nuclear accumulation of pSmad2 and EMT‐like response, indicating YAP1 activation is critical for pSmad2 to accumulate and activate transcription [9]. Taken together, the role of YAP/TAZ signaling is crucial in the TGFβ2‐induced EMT process.

MicroRNAs (miRNAs) have emerged as critical post‐transcriptional regulators of gene expression in ocular development and disease. While the miR‐200 family (miR‐200a, miR‐200b, miR‐200c, miR‐141, and miR‐429) is extensively studied in oncology—often acting as a tumor suppressor that inhibits EMT by targeting ZEB1/2 [10–13]—its function appears to be highly tissue dependent. Recent evidence suggests a paradox where miR‐200c can act as an oncogene or fibrosis promoter in specific contexts [14, 15].

Despite the known involvement of the miR‐200 family in cancer, the specific role of miR‐200c in the LECs remains unclear. Furthermore, whether miR‐200c modulates the YAP signaling pathway during PCO formation has not been elucidated. In this study, we investigated the expression profile of miR‐200c in TGFβ2‐induced LECs and explored its regulatory relationship with YAP, providing novel insights into the epigenetic regulation of PCO.

## 2. Materials and Methods

### 2.1. Cell Culture and Transfection

HLE B3 cells were purchased from the American Type Culture Collection (ATCC, Manassas, VA, USA), grown in Dulbecco’s modified Eagle’s medium/F12(1:1) (DMEM/F12, Hyclone, Beijing, China) supplemented with 20% premium fetal bovine serum (FBS) (Biological Industries, Israel), 50 U/mL of penicillin, and 50 μg/mL streptomycin (Hyclone, Beijing, China). Cells were maintained at 37°C in a humidified 5% CO_2_ atmosphere. The cells were transfected with miR‐200c‐3p agomir, miR‐200c‐3p antagomir, and miR‐200c‐3p agomir or antagomir negative control (NC) from GenePharma (Shanghai, China) using Lipofectamine 2000 (Invitrogen; Thermo Fisher Scientific, Inc.), following the manufacturer’s protocol. Sixteen hours after transfection, new DMEM/F12 medium with FBS changed in the presence or absence of TGFβ2 (10 ng/mL, R&D, USA).

### 2.2. Reverse Transcription‐Quantitative Polymerase Chain Reaction (RT‐qPCR) Analysis

Total RNA was extracted from the cultured cells using an RNA extraction kit (Tiangen, Beijing, China) according to the manufacturer’s protocol. To assess the expression levels of miR‐200c‐3p, cDNA was generated using specific reverse transcription primers (GenePharma, Shanghai, China) and M‐MLV reverse transcriptase (Promega, Madison, WI, USA). Small nuclear RNA U6 served as the reference for miRNA normalization. A total of 1 μg of RNA was reverse transcribed with PrimeScript™ RT Master Mix (Takara, Japan). Real‐time quantitative PCR was conducted utilizing SYBR Primix Ex Taq (Takara, Japan) and the StepOnePlus Real‐Time PCR system (Applied Biosystems, Carlsbad, CA, USA), following the manufacturer’s instructions. Thermocycling conditions were adopted from the protocol established by Wang et al. [16]. GAPDH was utilized as the internal control for mRNA, and relative expression was calculated using the 2^−ΔΔCT^ method.

### 2.3. Scratch Assay

The cells were inoculated in six‐well plates and routinely cultured until they reached 70%–80% confluence. Then, a scratch was made with a 200‐μL pipette tip and the cells were washed three times with sterile PBS to remove from the scratched region. The cells were continually cultured in a serum‐free culture medium with or without TGFβ2 (10 μg/mL as final concentration, R&D, USA) added into the culture medium. Images of the cells were captured at 0 and 48 h postscratching. To quantify migration, the wound area was measured using ImageJ software (NIH, Bethesda, MD, USA). The migration rate was calculated as follows:

(1) Wound closure %=area at 048 h−area at  harea at 0 h×100%.

### 2.4. Western Blot Analysis

HLE B3 cells were lysed in RIPA buffer (Beyotime Institute of Biotechnology, Shanghai, China), and proteins were harvested after centrifugation. Sample concentration was detected using the BCA protein assay kit (Thermo, Rockford, IL, USA). Approximately 30 μg of protein per sample were resolved by SDS‐PAGE and transferred to PVDF membranes. Membranes were blocked with 5% nonfat milk in PBST for 1.5 h at room temperature, followed by overnight incubation at 4°C with primary antibodies: anti‐GAPDH (1:4000 dilution, Abcam, Cambridge, MA, USA), anti‐vimentin (1:2000 dilution, Abcam, Cambridge, MA, USA), anti‐fibronectin (1:1000 dilution, Abcam, Cambridge, MA, USA), anti‐α‐SMA (1:1000 dilution, Abcam, Cambridge, MA, USA), anti‐E‐cadherin (1:1000 dilution, Abcam, Cambridge, MA, USA), anti‐Yap1 (1:1000 dilution, Abcam, Cambridge, MA, USA), and anti‐pYap (1:2000 dilution, Abcam, Cambridge, MA, USA), at 4°C overnight. After incubating with horseradish peroxidase‐conjugated goat anti‐rabbit IgG or goat anti‐mouse IgG (1:10,000 dilution, Abcam, Cambridge, MA, USA) for 1.5 h, an ECL + western blotting system kit (Abcam, Cambridge, MA, USA) was utilized for color development. The density of the bands was quantified using ImageJ software (Version 6.0; National Institutes of Health). GAPDH served as an internal control.

### 2.5. Immunofluorescence Staining

HLE B3 cells were seeded on coverslips and treated with miR‐200c‐3p agomir, miR‐200c‐3p antagomir, or controls. Cells were fixed with 4% paraformaldehyde for 20 min, permeabilized with 0.5% Triton X‐100, and blocked with 5% BSA. Cells were incubated with Plus 488‐conjugated anti‐YAP1 primary antibody (1:400 dilution, Proteintech, Rosemont, IL, USA) overnight at 4°C. DAPI (Beyotime, China) was used for nuclear counterstain for 5 min. Images were acquired using a fluorescence microscope (DM6B, Leica, Wetzlar, Germany).

### 2.6. Computational Target Prediction

Bioinformatics analysis software starBase v3.0 (https://starbase.sysu.edu.cn/index.php) was used to predict the target genes of miR‐200c‐3p [17, 18]. Results showed that miR‐200c‐3p could potentially bind to YAP1 (Supporting Table S1).

### 2.7. Statistical Analysis

All results are presented as mean ± S.E.M. from at least five independent biological replicates (*n* ≥ 5). This sample size was chosen based on previous similar in vitro studies in the field of LEC research, providing sufficient power to detect biologically meaningful differences without unnecessary use of resources. The study was designed around this prespecified number of experiments, and group sizes were equal across all experimental conditions.

An independent biological replicate is defined as a separate cell culture experiment using a different cell passage. Technical replicates (e.g., triplicate PCR wells, multiple image fields from a single coverslip, or repeated densitometric scans of the same blot) were averaged to provide a single value for each biological replicate and were not treated as independent *n* values.

**FIGURE 1:**
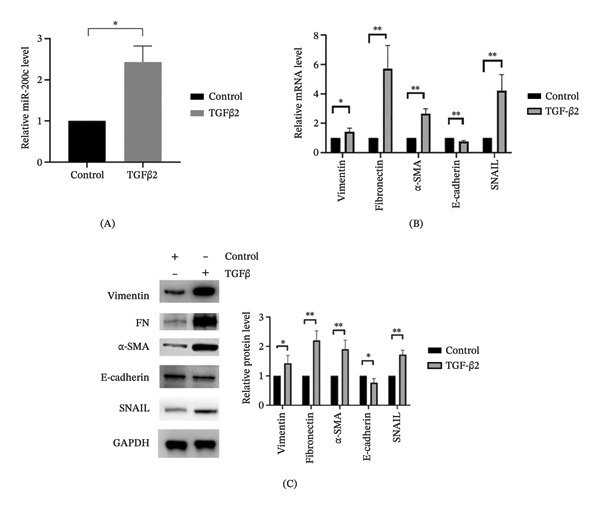
The expression levels of EMT markers and miR‐200c in TGFβ2‐treated human lens epithelial cells (LECs). (A) The expression level of miR‐200c was found to be upregulated in LECs treated with TGFβ2. (B) The mRNA expression levels of fibronectin, vimentin, α‐SMA, E‐cadherin, and SNAIL were assessed using real‐time quantitative PCR (RT‐qPCR), with GAPDH serving as the endogenous control. (C) The protein expression levels of fibronectin, vimentin, α‐SMA, E‐cadherin, and SNAIL were evaluated through western blot analysis. GAPDH served as an internal control. ^∗^ *p* < 0.05 and ^∗∗^ *p* < 0.01. Data represent *n* = 5 independent biological replicates. Error bars represent mean ± SEM. Statistical significance was determined by Student’s *t*‐test.

**FIGURE 2:**
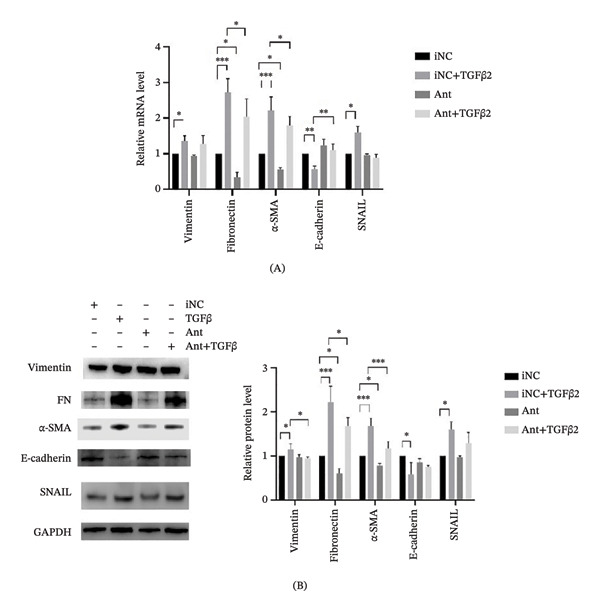
MiR‐200c antagomir inhibits LECs EMT. MiR‐200c antagomir (Ant) and antagomir negative control (iNC) were transfected into LECs in the presence or absence of TGFβ2 (10 ng/mL). (A) The relative mRNA levels of fibronectin, vimentin, α‐SMA, E‐cadherin, and SNAIL were assessed by RT‐qPCR, with GAPDH serving as the endogenous control. (B) The protein expression levels of fibronectin, vimentin,α‐SMA, E‐cadherin, and SNAIL were evaluated using western blot. GAPDH was utilized as a loading control. ^∗^ *p* < 0.05 and ^∗∗^ *p* < 0.01. Data represent *n* = 5 independent biological replicates. Error bars represent mean ± SEM. Statistical significance was determined by one‐way ANOVA followed by Tukey’s post hoc test.

**FIGURE 3:**
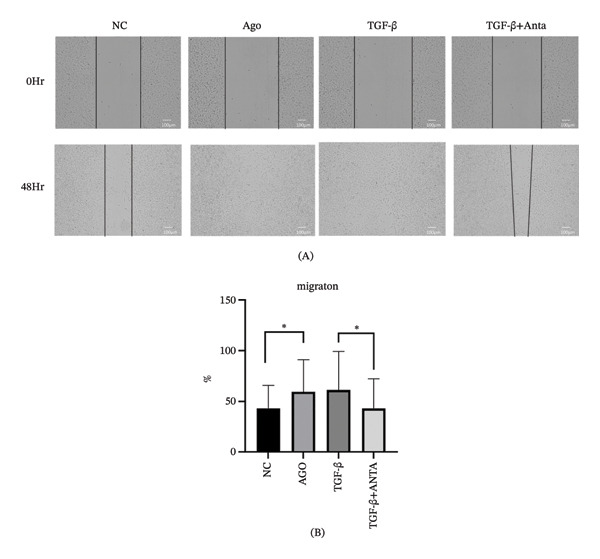
Expression of miR‐200c promotes LECs migration. (A) Image and (B) relative migration area comparison of the wound‐healing assay. ^∗^ *p* < 0.05. Data represent *n* = 6 independent biological replicates. Error bars represent mean ± SEM. Statistical significance was determined by one‐way ANOVA followed by Tukey’s post hoc test. NC, negative control; Ago, miR‐200c agomir; Anta, miR‐200c antagomir.

Before statistical analysis, the normality of the data was assessed using the Shapiro–Wilk test and further verified by visual inspection of Q–Q plots. Homogeneity of variance was confirmed by Levene’s test. Student’s *t*‐test was employed to evaluate differences between two means. For comparisons involving multiple means, one‐way ANOVA followed by Tukey’s test was conducted (specifically Figures 1–4). Tukey’s post hoc test was utilized for pairwise comparisons only after a significant overall ANOVA result was obtained (*P* < 0.05). All comparisons reported were preplanned. If assumptions of normality or equal variance had been violated, nonparametric alternatives (Mann–Whitney U or Kruskal–Wallis test) were planned; however, no data transformation or nonparametric testing was required for this study. No data points were excluded from the analysis, and no outliers were detected. All statistical analyses were carried out using GraphPad Prism (Version 9; GraphPad Software, Inc.). *p* values are reported as exact values where possible.

FIGURE 4MiR‐200c activates YAP in LECs. MiR‐200c agomir (Ago) and antagomir (Anta) were transfected into LECs in the presence or absence of TGFβ2 (10 ng/mL). YAP‐TEAD Inhibitor 1 (25 nM) was exclusively administered to the group treated with Ago and TGFβ2. (A) The protein expression levels of YAP were assessed using western blot. GAPDH served as a loading control. (B) The mRNA expression levels of YAP were assessed by RT‐qPCR. (C) The protein expression levels of fibronectin, vimentin, and SNAIL were evaluated through western blot, with GAPDH used as a loading control. (D) Treatment of MiR‐200c agomir for 24 h induced nuclear translocation of YAP. ^∗^ *p* < 0.05 and ^∗∗^ *p* < 0.01. Data represent *n* = 5 independent biological replicates. Error bars represent mean ± SEM. Statistical significance was determined by one‐way ANOVA followed by Tukey’s post hoc test.

**Figure:**
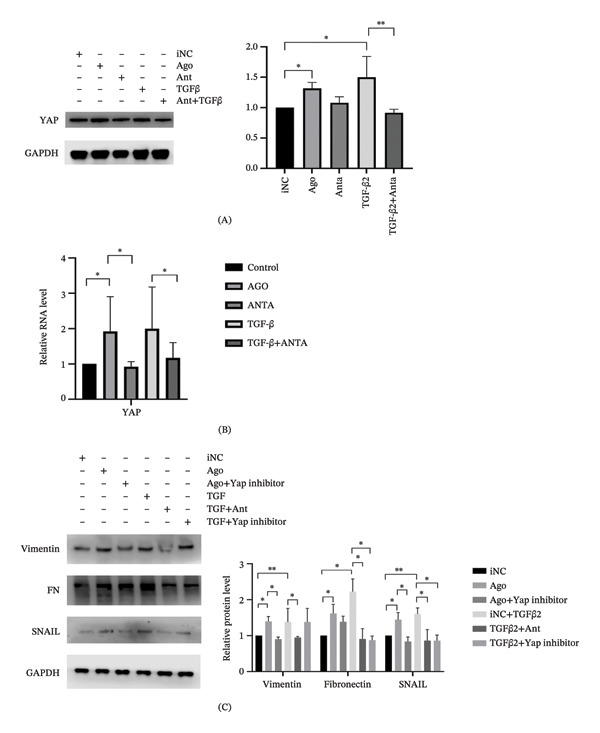

**Figure:**
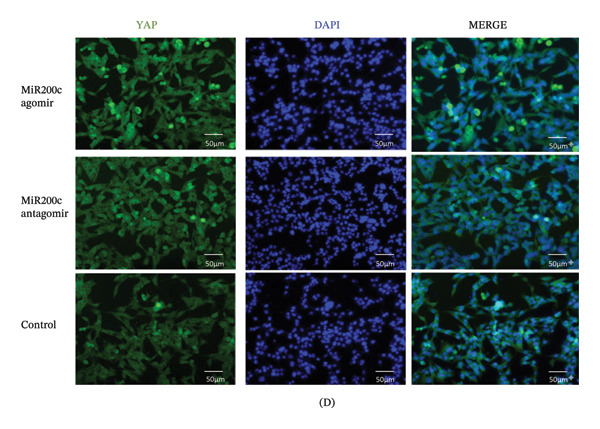

## 3. Results

### 3.1. MiR‐200c Was Upregulated in TGFβ2‐Treated LECs

To investigate the potential effects of miR‐200c in EMT of LECs, we applied TGFβ2 to induce EMT of LECs. The expression of miR‐200c was determined in LECs during the EMT process. It was shown in Figure 1A that the expression of miR‐200c was significantly upregulated in TGFβ2 treated LECs, increasing by approximately 2.43 ± 0.39 fold compared with control cells (*P* = 0.024). In addition, the EMT‐related factors in TGFβ2‐treated LECs were also determined. Expectedly, the expression of vimentin, fibronectin, and α‐SMA were all significantly enhanced and E‐cadherin was attenuated by TGFβ2. The mRNA levels of fibronectin, vimentin, SNAIL, and α‐SMA were enhanced by 5.71 ± 0.92, 1.42 ± 0.14, 4.02 ± 0.34, and 2.65 ± 0.20 fold, respectively (*p* values: fibronectin = 0.026, vimentin = 0.0012, SNAIL = 0.008, and α‐SMA = 0.0014), while E‐cadherin mRNA levels decreased to 0.76 ± 0.05 fold of the control (*P* = 0.0043) (Figure 1B and C).

### 3.2. MiR‐200c Promotes the EMT of LECs

**FIGURE 5:**
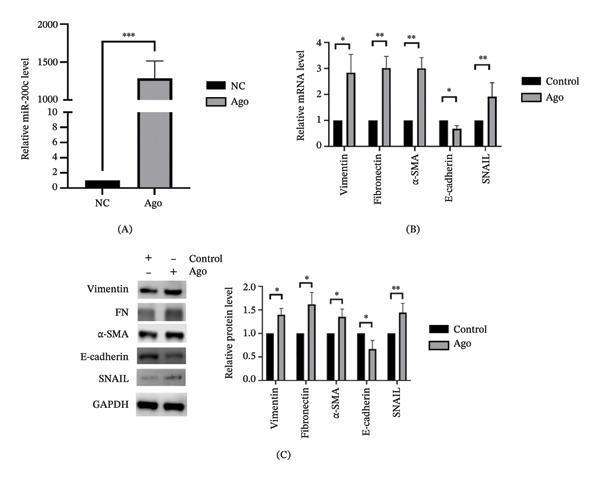
MiR‐200c agomir was transfected into LECs. (A) The relative expression of miR‐200c was assessed by RT‐qPCR, using RNA U6 as an endogenous control. (B) The mRNA expression levels of fibronectin, vimentin, α‐SMA, E‐cadherin, and SNAIL were measured via real‐time quantitative PCR (RT‐qPCR), with GAPDH serving as the endogenous control. (C) The protein expression levels of fibronectin, vimentin, α‐SMA, E‐cadherin, and SNAIL were evaluated through western blot analysis; GAPDH was utilized as a loading control. ^∗^ *p* < 0.05, ^∗∗^ *p* < 0.01, and ^∗∗∗^ *p* < 0.001. Data represent *n* = 5 independent biological replicates. Error bars represent mean ± SEM. Statistical significance was determined by Student′s *t*‐test.

To determine the roles of miR‐200c in TGFβ2‐treated LECs, miR‐200c agomir (Ago), miR‐200c antagomir (Anta), and miR‐200c NC were transfected into LECs, respectively. The results were verified by qRT‐PCR, that the miR‐200c expression was dramatically increased in LECs transfected with miR‐200c agomir by the fold of 1286.37 ± 132.00 (*p* < 0.00014) (Figure 5A). Furthermore, the overexpression of miR‐200c significantly upregulated the mRNA levels of mesenchymal markers; vimentin, fibronectin, SNAIL, and *α*‐SMA increased by 2.84 ± 0.40, 3.02 ± 0.26, 1.72 ± 0.22, and 3.01 ± 0.24 fold, respectively, compared with the NC group (*p* values: fibronectin = 0.0062, vimentin = 0.047, SNAIL = 0.0092, and α‐SMA = 0.0064) (Figure 5B). The protein levels mesenchymal markers were also upregulated accordingly ((*p* values: fibronectin = 0.026, vimentin = 0.037, SNAIL = 0.0054, and α‐SMA = 0.018)) (Figure 5C).

### 3.3. Transfection of MiR‐200c Antagomir Inhibits TGFβ2‐Induced EMT of LECs

qRT‐PCR was used to investigate the role of miR‐200c in EMT of LECs, and it revealed that the mRNA levels of vimentin, fibronectin, and α‐SMA were upregulated in the TGFβ2 group, whereas the E‐cadherin was downregulated compared with the control group (Figure 2A). After the transfection of miR‐200c antagomir, the effect of TGFβ2 seemed to be attenuated, for lower mRNA levels of fibronectin and α‐SMA and higher level of E‐cadherin were observed than those in TGFβ2‐treated cells (*p* values: fibronectin = 0.038, α‐SMA = 0.014, and E‐cadherin = 0.0065) (Figure 2A). Western blot results further confirmed the result, significantly higher protein levels of vimentin, fibronectin, and α‐SMA were observed in the TGFβ2‐treated cells. This upregulation trend can be reduced with the transfection of MiR‐200c antagomir (*p* values: vimentin = 0.029, fibronectin = 0.026, and α‐SMA = 0.00035) (Figure 2B). Collectively, these findings suggest that the overexpression of miR‐200c antagomir can significantly inhibit TGFβ2‐induced EMT in LECs.

### 3.4. Expression of MiR‐200c Promotes LEC Migration

To further investigate the effect of miR‐200c on LECs mobility in vitro, the wound‐healing assay was performed. As the results showed, the LECs transfected with miR‐200c agomir (Ago) were more migratory than the NC cells. TGFβ2 also promoted the LEC migration, which can be diminished after being transfected with miR‐200c antagomir. As shown in Figure 3, quantitative analysis of the wound‐healing assay revealed that the relative migration rate in the miR‐200c agomir group was 59.45 ± 14.19%, significantly higher than the 42.93 ± 10.22% observed in the NC group (*P* = 0.032). These observations suggested that miR‐200c is important in increasing the migration of LECs in vitro (Figure 3A,B).

### 3.5. MiR‐200c Promotes EMT of LECs via Regulating YAP Expression and Nuclear Translocation

To unravel the possible mechanism of miR‐200c in mediating EMT, we investigated the relationship between miR‐200c and YAP. Although the bioinformatics software starBase predicts potential binding sites between miR‐200c and YAP mRNA (Supporting Table S1), canonical miRNA–mRNA interaction typically results in gene suppression. However, our RT‐qPCR and Western blot analyses revealed that YAP expression was significantly upregulated in both the TGFβ2‐treated group and the miR‐200c agomir‐transfected group (*p* values: Ago = 0.048 and TGFβ2 = 0.033) (Figure 4A) (*p* values: Ago = 0.025) (Figure 4B). To further confirm YAP activation, immunofluorescence staining was performed to visualize YAP subcellular localization (Figure 4D). In control cells, YAP was predominantly cytoplasmic. Upon miR‐200c agomir transfection, we observed a marked increase in nuclear accumulation of YAP, indicating that miR‐200c facilitates the nuclear translocation of YAP.

To further elucidate the role of Yap in EMT in LECs, the YAP‐TEAD Inhibitor 1 (peptide 17) (25 nM) (purchased from Selleck Chemicals, Shanghai, China) was added to the cell culture. As shown in Figure 4C, the pro EMT effect (upregulation of EMT markers, including fibronectin, vimentin, and SNAIL) induced by miR‐200c agomir in LECs was relieved by the emergence of YAP‐TEAD Inhibitor 1 (*p* values: Vimentin Ago + YAP inhibitor = 0.036 and SNAIL Ago + YAP inhibitor = 0.043). These findings demonstrate that blockade of the Yap‐associated pathway can effectively diminish the promoting effect of miR‐200c agomir on EMT.

## 4. Discussion

PCO remains a significant concern for postsurgery visual impairment. It is characterized by wound‐healing response and fibrosis processes, where LECs exhibit proliferation and migration and undergo EMT [4]. Therefore, inhibiting this process holds great significance for its treatment. In this study, we assessed the transcriptional activity of the miR‐200 family within LECs and identified a pivotal role for miR‐200c. Remarkably, upon exposure to TGFβ2 treatment, we observed a substantial upregulation of miR‐200c, which proved essential for promoting the EMT process.

The role of the miR‐200 family has been extensively debated in cell biology. Classically, they are described as the keepers of the epithelial phenotype, inhibiting EMT by direct inhibition of the transcriptional repressors ZEB1 and ZEB2 [10, 19]. In several cancer models, such as nonsmall cell lung cancer and prostate cancer, the downregulation of miR‐200c is associated with metastasis and drug resistance [20, 21]. However, recent studies have reported an increase in miR‐200c‐3p expression in cancer tissue, along with its promotion of cell viability, proliferation, migration, invasion, and EMT in gastric cell carcinoma, papillary thyroid carcinoma, and serous ovarian cancer [15, 22, 23]. Furthermore, ZEB1‐independent regulation has been identified including the study of Posch et al., which is characterized by the upregulation of miR‐200c associated with a poor prognosis, regardless of the level of ZEB1 expression [14]. Our findings in human LECs align with this noncanonical profile. We observed that high levels of miR‐200c correlated with increased expression of EMT markers (fibronectin, vimentin, and α‐SMA). On the other hand, the inhibition of miR‐200c using an antagomir was able to restore the epithelial phenotype despite TGFβ2, suggesting that miR‐200c acts as a profibrotic agent in this particular biological context.

A major finding of this study is the identification of the downstream mechanism by which miR‐200c drives EMT: the activation of the YAP signaling pathway, while bioinformatics tools (starBase) predicted a potential binding site between miR‐200c and YAP mRNA (Supporting Table S1), where miRNA–mRNA interactions typically result in gene silencing. Intriguingly, we observed a positive correlation that the overexpression of miR‐200c resulted in higher levels of both YAP mRNA and protein, thus suggesting that miR‐200c does not degrade YAP directly but more likely regulating it indirectly, probably through the inhibition of upstream negative regulators of the Hippo pathway.

Crucially, our immunofluorescence results highlighted that miR‐200c not only increases total YAP levels but also greatly enhances YAP nuclear translocation. In hair follicle–related research, it has been observed that the miR‐200 family exerts its influence on the Hippo pathway by inhibiting the phosphorylation of downstream effector protein YAP [24]. Phosphorylated YAP accumulates in the cytoplasm and is subjected to rapid degradation through ubiquitination and lysosomal pathways [25]. Nonphosphorylated YAP, on the other hand, translocates into the nucleus, where it interacts with transcription factor TEAD family members to activate the transcription of target genes, ultimately promoting cell proliferation and EMT. Consistent with these findings, our study indicated that miR‐200c agomir could increase nuclear YAP accumulation, implying that miR‐200c may interfere with the phosphorylation machinery that normally keeps YAP in check. With the specific YAP‐TEAD Inhibitor 1 (peptide 17), we were able to reverse the pro‐EMT effect of miR‐200c, demonstrating that the nuclear activity of YAP was the key downstream effector of miR‐200c in LECs.

The relevance of the YAP pathway to lens pathology is supported by recent literature. One study revealed that the incubation of rat lens explants with verteporfin, a YAP inhibitor, effectively mitigated the TGFβ2‐induced development of a fiber‐like phenotype, as well as the expression of α‐SMA and fibronectin. Furthermore, it successfully prevented the delocalization of E‐cadherin and *β*‐catenin [26]. Additionally, elevated level of TGFβ2 in the aqueous humor is a known risk factor for cataract formation [27]. Our results connect these findings by placing miR‐200c at the center of an epigenetic regulatory pathway, which potentiates the TGFβ2/YAP axis. Upregulation of YAP and promoting its nuclear localization by miR‐200c allow the enhanced expression of SNAIL and vimentin in the process of EMT in LECs.

Further investigation is required to determine the precise downstream signaling pathways. While bioinformatics tools predicted a potential interaction, our data consistently showed that miR‐200c overexpression leads to increased YAP levels. This suggests miR‐200c is acting as an upstream activator of YAP signaling in LECs, probably via indirect regulation, instead of direct canonical repression.

In summary, our findings provide compelling evidence supporting the involvement of the miR‐ 200c/Yap pathway in TGFβ2‐induced EMT of LECs. Notably, transfection with the agomir sequence of miR‐200c significantly promotes EMT, while the inhibition of miR‐200c effectively prevents TGF‐induced EMT from occurring. However, this study has several limitations. First, all the experiments were conducted exclusively in vitro on HLE‐B3 cell and it is necessary to validate on an in vivo animal model or with real human capsular tissues in physiological lens environment. Second, although demonstrated miR‐200c upregulates YAP, it is yet to be determined what would be the direct target of miR‐200c in the activation of YAP. Future research should focus on identifying the specific upstream negative regulators of the Hippo pathway targeted by miR‐200c.

## Author Contributions

Yin‐juan Wei, Yi‐an‐zhu Liu, and Ying Dai contributed equally to this study.

## Funding

This study was funded by Tianjin Eye Hospital Science and Technology Fund General Project (YKYB2002) and Tianjin Key Medical Discipline (Specialty) Construction Project (no. TJYXZDXK‐016A). This study was also supported by Nankai University Eye Institute (NKYKK202211) and National Natural Science Foundation of China (Grant no. 62272248).

## Disclosure

We confirm that this manuscript has not been published elsewhere and is not under consideration by another journal. All authors have approved the manuscript and agree with submission to Journal of Ophthalmology.

## Conflicts of Interest

The authors declare no conflicts of interest.

## Supporting Information

Additional supporting information can be found online in the Supporting Information section.

## Supporting information


**Supporting Information** Bioinformatics analysis software starBase v3.0 (https://starbase.sysu.edu.cn/index.php) was utilized to predict the target genes of miR‐200c‐3p [21, 22]. The results indicated that miR‐200c‐3p has the potential to bind to YAP1 (Supporting Table S1).

## Data Availability

The datasets used and/or analyzed during the current study are available from the corresponding author upon reasonable request.

## References

[1] Konopinska J. , Mlynarczyk M. , Dmuchowska D. A. , and Obuchowska I. , Posterior Capsule Opacification: A Review of Experimental Studies, Journal of Clinical Medicine. (2021) 10, no. 13, 10.3390/jcm10132847. PMC8269180 34199147

[2] Zhang X. , Wang J. , Xu J. et al., Prophylaxis of Posterior Capsule Opacification Through Autophagy Activation With Indomethacin-Eluting Intraocular Lens, Bioactive Materials. (2023) 23, 539–550, 10.1016/j.bioactmat.2022.11.024. 36514385 PMC9729928

[3] Zhang R. P. and Xie Z. G. , Research Progress of Drug Prophylaxis for Lens Capsule Opacification After Cataract Surgery, J Ophthalmol.(2020) 2020, 2181685–2181689, 10.1155/2020/2181685. 32714607 PMC7355348

[4] Wormstone I. M. , Wang L. , and Liu C. S. , Posterior Capsule Opacification, Experimental Eye Research. (2009) 88, no. 2, 257–269, 10.1016/j.exer.2008.10.016. 19013456

[5] de Iongh R. U. , Wederell E. , Lovicu F. J. , and McAvoy J. W. , Transforming Growth Factor-Beta-Induced Epithelial-Mesenchymal Transition in the Lens: A Model for Cataract Formation, Cells Tissues Organs. (2005) 179, no. 1-2, 43–55, 10.1159/000084508. 15942192

[6] Sabbineni H. , Verma A. , and Somanath P. R. , Isoform-Specific Effects of Transforming Growth Factor Beta on Endothelial-to-Mesenchymal Transition, Journal of Cellular Physiology. (2018) 233, no. 11, 8418–8428, 10.1002/jcp.26801. 29856065 PMC6415927

[7] Noguchi S. , Saito A. , and Nagase T. , YAP/TAZ Signaling as a Molecular Link Between Fibrosis and Cancer, International Journal of Molecular Sciences. (2018) 19, no. 11, 10.3390/ijms19113674. PMC6274979 30463366

[8] Pefani D. E. , Pankova D. , Abraham A. G. et al., TGF-Beta Targets the Hippo Pathway Scaffold RASSF1A to Facilitate YAP/SMAD2 Nuclear Translocation, Molecular Cell. (2016) 63, no. 1, 156–166, 10.1016/j.molcel.2016.05.012. 27292796

[9] Gao Y. , Fan S. , Li H. et al., Constitutive Androstane Receptor Induced-Hepatomegaly and Liver Regeneration Is Partially via Yes-Associated Protein Activation, Acta Pharmaceutica Sinica B. (2021) 11, no. 3, 727–737, 10.1016/j.apsb.2020.11.021. 33777678 PMC7982502

[10] Korpal M. and Kang Y. , The Emerging Role of miR-200 Family of Micrornas in Epithelial-Mesenchymal Transition and Cancer Metastasis, RNA Biology. (2008) 5, no. 3, 115–119, 10.4161/rna.5.3.6558. 19182522 PMC3532896

[11] Lu Y. B. , Hu J. J. , Sun W. J. , Duan X. H. , and Chen X. , Prognostic Value of miR-141 Downregulation in Gastric Cancer, Genetics and Molecular Research. (2015) 14, no. 4, 17305–17311, 10.4238/2015.December.16.31. 26681225

[12] Yao Y. , Hu J. , Shen Z. et al., MiR-200b Expression in Breast Cancer: A Prognostic Marker and Act on Cell Proliferation and Apoptosis by Targeting Sp1, Journal of Cellular and Molecular Medicine. (2015) 19, no. 4, 760–769, 10.1111/jcmm.12432. 25639535 PMC4395190

[13] Zhu Z. M. , Xu Y. F. , Su Q. J. et al., Prognostic Significance of microRNA-141 Expression and Its Tumor Suppressor Function in Human Pancreatic Ductal Adenocarcinoma, Molecular and Cellular Biochemistry. (2014) 388, no. 1-2, 39–49, 10.1007/s11010-013-1897-y. 24242138

[14] Posch F. , Prinz F. , Balihodzic A. et al., MiR-200c-3p Modulates Cisplatin Resistance in Biliary Tract Cancer by ZEB1-Independent Mechanisms, Cancers (Basel). (2021) 13, no. 16, 10.3390/cancers13163996. PMC8392278 34439151

[15] Feng Y. L. , Ke T. , Wang G. L. , Qi H. Y. , and Xiao Y. , MicroRNA-200c-3p Negatively Regulates ATP2A2 and Promotes the Progression of Papillary Thyroid Carcinoma, Biochemical Genetics. (2022) 60, no. 5, 1676–1694, 10.1007/s10528-022-10184-w. 35079913 PMC8788908

[16] Wang S. J. , Li W. W. , Wen C. J. , Diao Y. L. , and Zhao T. L. , MicroRNA-214 Promotes the EMT Process in Melanoma by Downregulating CADM1 Expression, Molecular Medicine Reports. (2020) 22, no. 5, 3795–3803, 10.3892/mmr.2020.11446. 33000202 PMC7533494

[17] Zheng T. , Chen W. , Wang X. , Cai W. , Wu F. , and Lin C. , Circular RNA circ-FAM158A Promotes Retinoblastoma Progression by Regulating miR-138-5p/SLC7A5 Axis, Experimental Eye Research. (2021) 211, 10.1016/j.exer.2021.108650. 34102206

[18] Wang S. , Diao Y. J. , and Zhu B. B. , MiR-193a-5p Suppresses Cell Proliferation and Induces Cell Apoptosis by Regulating HOXA7 in Human Ovarian Cancer, Neoplasma. (2020) 67, no. 4, 825–833, 10.4149/neo_2020_190730N687. 32305054

[19] Chen H. , Li Z. , Zhang L. et al., MicroRNA-200c Inhibits the Metastasis of Triple-Negative Breast Cancer by Targeting ZEB2, an Epithelial-Mesenchymal Transition Regulator, Annals of Clinical Laboratory Science. (2020) 50, no. 4, 519–527. 32826250

[20] Wang H. Y. , Liu Y. N. , Wu S. G. et al., MiR-200c-3p Suppression Is Associated With Development of Acquired Resistance to Epidermal Growth Factor Receptor (EGFR) Tyrosine Kinase Inhibitors in EGFR Mutant Non-Small Cell Lung Cancer via a Mediating Epithelial-to-Mesenchymal Transition (EMT) Process, Cancer Biomarkers. (2020) 28, no. 3, 351–363, 10.3233/CBM-191119. 32417760 PMC12662365

[21] Xia L. , Han Q. , Chi C. et al., Transcriptional Regulation of PRKAR2B by miR-200b-3p/200c-3p and XBP1 in Human Prostate Cancer, Biomedicine & Pharmacotherapy. (2020) 124, 10.1016/j.biopha.2020.109863. 31986411

[22] Wang Y. , Lu K. , Li W. et al., MiR-200c-3p Aggravates Gastric Cell Carcinoma via KLF6, Genes Genomics. (2021) 43, no. 11, 1307–1316, 10.1007/s13258-021-01160-6. 34524611 PMC8478742

[23] Ankasha S. J. , Shafiee M. N. , Abdul Wahab N. , Raja Ali R. A. , and Mokhtar N. M. , Oncogenic Role of miR-200c-3p in High-Grade Serous Ovarian Cancer Progression via Targeting the 3′-Untranslated Region of DLC1, International Journal of Environmental Research and Public Health. (2021) 18, no. 11, 10.3390/ijerph18115741. PMC8198916 34071861

[24] Hoefert J. E. , Bjerke G. A. , Wang D. , and Yi R. , The microRNA-200 Family Coordinately Regulates Cell Adhesion and Proliferation in Hair Morphogenesis, The Journal of Cell Biology. (2018) 217, no. 6, 2185–2204, 10.1083/jcb.201708173. 29602800 PMC5987720

[25] Zhang H. , Liu C. Y. , Zha Z. Y. et al., TEAD Transcription Factors Mediate the Function of TAZ in Cell Growth and Epithelial-Mesenchymal Transition, Journal of Biological Chemistry. (2009) 284, no. 20, 13355–13362, 10.1074/jbc.M900843200. 19324877 PMC2679435

[26] Taiyab A. , Belahlou Y. , Wong V. et al., Understanding the Role of Yes-Associated Protein (YAP) Signaling in the Transformation of Lens Epithelial Cells (EMT) and Fibrosis, Biomolecules. (2023) 13, no. 12, 10.3390/biom13121767. PMC10741558 38136638

[27] Jampel H. D. , Roche N. , Stark W. J. , and Roberts A. B. , Transforming Growth Factor-Beta in Human Aqueous Humor, Current Eye Research. (1990) 9, no. 10, 963–969, 10.3109/02713689009069932. 2276273

